# Upregulation of Programmed Death-1 and Its Ligand in Cardiac Injury Models: Interaction with GADD153

**DOI:** 10.1371/journal.pone.0124059

**Published:** 2015-04-22

**Authors:** Babak Baban, Jun Yao Liu, Xu Qin, Neal L. Weintraub, Mahmood S. Mozaffari

**Affiliations:** 1 Department of Oral Biology, College of Dental Medicine, Georgia Regents University, Augusta, Georgia 30912, United States of America; 2 Department of Medicine, Section of Cardiology and the Vascular Biology Center, Medical College of Georgia, Georgia Regents University, Augusta, Georgia 30912, United States of America; University of Southern California, UNITED STATES

## Abstract

**Purpose:**

Programmed Death-1 (PD-1) and its ligand, PD-L1, are regulators of immune/ inflammatory mechanisms. We explored the potential involvement of PD-1/PD-L1 pathway in the inflammatory response and tissue damage in cardiac injury models.

**Experimental Design:**

Ischemic-reperfused and cryoinjured hearts were processed for flow cytometry and immunohistochemical studies for determination of cardiac PD-1 and PD-L1 in the context of assessment of the growth arrest- and DNA damage-inducible protein 153 (GADD153) which regulates both inflammation and cell death. Further, we explored the potential ability of injured cardiac cells to influence proliferation of T lymphocytes.

**Results:**

The isolated ischemic-reperfused hearts displayed marked increases in expression of PD-1 and PD-L1 in cardiomyocytes; however, immunofluorescent studies indicate that PD-1 and PD-L1 are not primarily co-expressed on the same cardiomyocytes. Upregulation of PD-1/PD-L1 was associated with a) marked increases in GADD153 and interleukin (IL)-17 but a mild increase in IL-10 and b) disruption of mitochondrial membrane potential (ψ_m_) as well as apoptotic and necrotic cell death. Importantly, while isotype matching treatment did not affect the aforementioned changes, treatment with the PD-L1 blocking antibody reversed those effects in association with marked cardioprotection. Further, ischemic-reperfused cardiac cells reduced proliferation of T lymphocytes, an effect partially reversed by PD-L1 antibody. Subsequent studies using the cryoinjury model of myocardial infarction revealed significant increases in PD-1, PD-L1, GADD153 and IL-17 positive cells in association with significant apoptosis/necrosis.

**Conclusions:**

The data suggest that upregulation of PD-1/PD-L1 pathway in cardiac injury models mediates tissue damage likely through a paracrine mechanism. Importantly, inhibition of T cell proliferation by ischemic-reperfused cardiac cells is consistent with the negative immunoregulatory role of PD-1/PD-L1 pathway, likely reflecting an endogenous cardiac mechanism to curtail the deleterious impact of infiltrating immune cells to the damaged myocardium. The balance of these countervailing effects determines the extent of cardiac injury.

## Introduction

The immune system has evolved to distinguish and defend against foreign antigens while simultaneously avoiding self-reactivity. Protection against self-reactivity is primarily relegated to central tolerance mechanisms which cause depletion of most self-reactive T lymphocytes. Yet, some T lymphocytes that are specific for self-antigens escape into the periphery, which likely underlies the evolution of peripheral tolerance mechanisms in order to protect against autoimmunity. An important mechanism regulating peripheral tolerance and autoimmunity is the expression of the programmed death-1 (PD-1) receptor [1–3].

The PD-1 receptor is a coinhibitory member of the B7/CD28 superfamily of molecules which is expressed on T and B lymphocytes. Binding of PD-1 with ligand partners, namely PD-L1 and PD-L2, results in the upregulation of the suppressive arm of immunity, thereby protecting against self and microbial antigens [1]. PD-L1 is broadly expressed on hematopoietic and non-hematopoietic cells while PD-L2 expression is believed to be restricted primarily to macrophages and dendritic cells [1]. PD-1 is described as a mediator of CD28+ T cell exhaustion in chronic viral infection and cancer [4–6]. Indeed, a monoclonal PD-1 blocking antibody is in clinical trials for cancer [7]. Further, the programmed death pathway was shown to regulate inflammation in various disease settings including atherosclerosis, allograft vascular disease, encephalomyelitis, stroke, sepsis and viral myocarditis [8–15]. Importantly, however, the potential role of programmed death pathway in cardiac ischemia-reperfusion (IR) injury and myocardial infarction has not been explored.

Myocardial injury is associated with upregulation of endogenous inflammatory mechanisms [16–19]. An important emerging mechanism relates to the expression of the growth arrest- and DNA damage-inducible protein 153 (GADD153) which regulates cardiac inflammation and apoptosis [16–20]. Whether and how the PD-1/PD-L1 pathway might interact with GADD153 in the setting of cardiac IR injury and infarction is unclear. It is plausible that cardiac PD-1/PD-L1 might curtail the pro-inflammatory component of GADD153 expression thereby limiting tissue injury. Alternatively, it is possible that the PD-1/PD-L1 pathway promotes cardiomyocyte death in the damaged heart, as has been reported in the case of T cell apoptosis [1], likely through a mechanism involving GADD153 expression. To distinguish among these possibilities, we tested the hypothesis that upregulation of cardiac PD-1/PD-L1 pathway represents an important endogenous mechanism determining the outcome of an insult to the heart. These studies utilized the Langendorff-perfused heart subjected to an IR insult in the absence and presence of a PD-L1 blocking antibody in order to establish the impact of disruption of PD-1/PD-L1 signaling in the heart. Upon demonstration of marked upregulation of PD-1/PD-L1 in ischemic-reperfused cardiac cells, subsequent studies utilized the mixed lymphocytic reaction assay to determine whether these cells influence the proliferative capacity of T lymphocytes [21,22]. Finally, to establish the *in vivo* relevance of PD-1/PD-L1 pathway in cardiac injury, additional studies utilized the cryoinjury model of myocardial infarction [23,24].

## Materials and Methods

All animal procedures were performed in accordance with the approval of the Institutional Animal Care and Use Committee of the Georgia Regents University. No human subject was involved in this study.

Male Sprague-Dawley rats (9–11 weeks of age) and male BALB/c mice (11–12 weeks of age) were obtained from Harlan Laboratories (Indianapolis, IN) and housed in a room maintained at constant humidity (60 ± 5%), temperature (24 ± 1°C) and light cycle (0600–1800h).

For isolated heart perfusion experiments, rats were heparinized (1000 U/ kg) and decapitated prior to removing the hearts and perfusing them on a Langendorff apparatus [16–18]. The perfusion medium was standard Krebs-Henseleit buffer (37°C) containing 11 mM glucose and equilibrated with 95% O_2_-5% CO_2_; the perfusion pressure was set at 120 cmH_2_O. Following a 25-min period of stabilization, the hearts were subjected to 40 min of ischemia followed by 15 min of reperfusion; time-controlled normoxic hearts served as controls (n = 6 hearts/group). Prior to induction of the ischemic phase, some hearts were infused with a PD-L1 blocking antibody [n = 6; 20 μg/heart; eBioscience (cat# 16–5983), San Diego, CA] while control ischemic-reperfused hearts received isotype matching antibody [n = 4; 20 μg/heart; eBioscience (cat# 14-4714-82), San Diego, CA] via a side arm placed above the heart. Thereafter, cardiac tissue was filtered through a cell strainer (BD Biosciences, Bedford, MA) and centrifuged (1500 rpm, 10 minutes) to obtain single cell suspension for flow-cytometry based assays or cytospin-preparations for immunofluorescent studies. Also, myocardial tissue was fixed in buffered formalin for subsequent immunohistochemical studies [16–18].

*In vivo* studies utilized the cryoinjury model of myocardial infarction which produces standardized infarction size [23,24]. Accordingly, male BALB/c mice were subjected to either cryoinjury (n = 7) or sham operation (n = 4). In preparation for the surgical procedure, each animal was anesthetized with ketamine/xylazine (120/16 mg/kg; i.p.) followed by tracheal intubation and ventilation using a mechanical ventilator. Thereafter, the heart was exposed through a left lateral thoracotomy and opening of the pericardium. Cryoinfarction was produced by application of a 4-mm diameter, liquid nitrogen-cooled, metal probe for 10 seconds to the anterior left ventricular wall, midway between an imaginary line from the left atrium to the apex of the heart; sham animals underwent a similar procedure but the metal probe was at room temperature. Three hours after the procedure, the hearts were harvested and cells prepared for flow cytometry protocols as described above [16–18].

### Analytical Flow Cytometry

Flow cytometry offers the distinct advantage of determination of multiple parameters using the same pool of cells from a given tissue [16–17]. Further, assessment of protein levels using flow cytometry is consistent with their determination using Western blotting [17]. Accordingly, commercially available antibodies against each protein of interest were used coupled with the use of a *FACSCalibur* flow cytometer (BD BioSciences, San Diego, CA) as described previously [16–18]. Briefly, the primary antibodies against PD-1 (cat no. ab36151), isotype control (cat no. ab37374) and PD-L1 (cat no. ab174838) and isotype control (cat no. 172730) were purchased from abcam Biotech. On the other hand, IL-17 and IL-10 were purchased from affymetrix ebioscience (IL-17 anti rat/mouse cat no. 53-7177-81, isotype control cat no. 53–4321) and BD BioSciences (IL-10 anti rat cat no. 555–088, isotype control cat no. 555–058); antibody against GADD153 was purchased from biorbyt (Anti-GADD153 cat no. arb15638; isotype control from abcam, cat no. 172730). As a gating strategy, for each sample, isotype-matched controls were analyzed to set the appropriate gates; representative data are reported in relevant figures. For each marker, samples were analyzed in duplicate measurements. To minimize false-positive events, the number of double-positive events detected with the isotype controls was subtracted from the number of double-positive cells stained with corresponding antibodies (not isotype control), respectively. Cells expressing a specific marker were reported as a percentage of the number of gated events.

### Immunostaining

#### A) Immunofluorescence staining

Tissue sections (5 μm) were prepared from formalin-fixed paraffin-embedded tissues. Following de-paraffinization, sections were washed for 10 min in distilled water. To permeabilize, all preparations were incubated in 0.2% Triton X-100 for 5 min at room temperature. All slides were washed three times, each time for 5 min, at room temperature and then incubated in blocking buffer (20% normal donkey serum, 1% BSA, 0.02% NaN3, 1× PBS) for 45–60 min. Following treatment with the primary antibody [anti-brain natriuretic peptide (BNP) antibody (Cardiomyocyte marker (cat no. ab47685), anti PD-1 (cat no. ab36151), anti PD-L1 (cat no. ab174838); abcam, USA] overnight at 4°C, preparations were then washed three times with Tris-buffered saline (TBS) for 5 min each time. All slides were then incubated with the secondary fluorescence-labeled antibody (1:100, catalog no 111–165003 and 711-545-152; Jackson Immunoresearch Laboratories, West Grove, PA, USA) for 1 hour in the dark at room temperature, washed twice in TBS for 5 min each time in the dark and then counterstained using DAPI (4',6-Diamidino-2-Phenylindole, Dihydrochloride; catalog no. D-1306, Life Technologies, USA). Further, cytospin preparations of about 20, 000 sorted cells per sample chamber were centrifuged (700 r.p.m., 5 min), air-dried, fixed in 10% formalin and washed twice in PBS. All cytospin preparations were then treated as described above. Microscopic analysis and imaging was done by an Olympus BX40 using Q-Capture software.PBS.

#### B) Immunohistochemistry

Tissue sections were prepared as described above. Following de-paraffinization, sections were washed for 10 min in distilled water. All subsequent procedures with tissue preparations were carried out at room temperature [21,22]. Endogenous peroxidase activity was blocked with hydrogen peroxide (1:10 w/PBS, 10 min). Thereafter tissue sections were treated with proteinase K (catalog no. S3020; DAKO, Carpentaria, CA, USA) for 10 min. After two washes in PBS, tissue preparations were treated with TritonX for 15 minutes, rinsed in PBS and incubated with anti-Caspase 3 antibody (1:100 in PBS; cat no. ab17815 abcam). After two washes in PBS, preparations were treated with biotinylated goat anti-rabbit Ig (catalog no. HK336-9R, BioGenex). Following a 5-min wash in PBS, slides were incubated for 20 min in peroxidase-conjugated streptavidin (catalog no. HK330-9k, BioGenex). Caspase 3 cells were visualized using 3-amino-9-ethylcarbazole chromogen (catalog no. HK121-5K Liquid AEC, BioGenex) for 3–5 minutes for optimal staining. Preparations were counterstained with hematoxylin (catalog no. 7221; Richard-Allan Scientific, Kalamazoo, MI, USA) and mounted in Faramount (catalog no. S3025, DAKO).

### Assessment of Mitochondrial Membrane Potential (ψ_m_)

As described previously [16–18], flow cytometry-based JC-1 assay was used to assess ψ_m_ as a surrogate marker of mitochondrial permeability transition (MPT) pore opening, which is a critical event in cell death.

### Assessment of Cell Death

Assessment of necrosis and apoptosis utilized the flow-cytometry-based annexin V/7-Amino-Actinomycin D (7-AAD) protocol as described previously [16–18]. Annexin V is used as a marker of apoptosis and 7-AAD is a standard flow cytometric viability probe which is used to distinguish viable from nonviable cells. Staining was performed according to the manufacturer's instructions [Annexin V: PE Apoptosis Detection Kit; BD Biosciences (Bedford, MA)]. Further assessment of cell death was carried out using caspase 3 immunohistochemistry as described above.

### Magnetic Assorted Cell Sorting (MACS)

T cells (CD3+) enrichment was performed using CD3 MicroBeads (catalog no. 130-052-001; Miltenyi Biotec, Auburn, USA) as described previously [21]. Cell separation with the MACS Separator was carried out according to the manufacturer instructions. Briefly, between 500 μL and 1 mL of cell suspension in RPMI culture medium were used for enrichment. Cells were magnetically-labeled by adding 50 μL MicroBeads per 1 mL of suspension. Cells were incubated at 4–8°C for 15 min and washed subsequently by adding 5 mL of MACS Running Buffer (Miltenyi Biotec) per 1 mL of PB. Cells were centrifuged and resuspended in one volume of buffer. For obtaining higher purity, the whole process was repeated twice. After two purification steps, the positive fraction containing CD3+ cells (85% purity) was used in the mixed lymphocytic reaction.

### Mixed Lymphocytic Reaction (MLR)

The MLR is a commonly used functional assay to determine the proliferative capacity of T lymphocytes in response to antigen presentation [21,22]. This assay requires the use of responder cells (e.g., splenic T cells) and stimulating cells (e.g., antigen presenting cells (APCs) or cardiac cells) from animals with different genetic backgrounds. The responder cells (i.e., splenic T cells of Wistar-Kyoto rats) and stimulators cells (i.e., normoxic and ischemic-reperfused cardiac cells of Sprague-Dawley rats) were set up in triplicate wells in a RPMI 1640 medium which was supplemented with fetal bovine serum, penicillin, streptomycin, L-glutamine and 2-mercaptoethanol. To examine the role of PD-1/PD-L1 in this functional assay, the PD-L1 blocking antibody was included in some experiments; APCs were included as internal control [21,22]. Responder T cells were initially enriched using Magnetic Assorted Cell Sorting (MACS) and used at 1x10^5^ cells/well. Cardiac cells or APCs (as stimulators) were used at 5 x10^5^/well. Following 72–96 hours of incubation at 37°C in a humidified 5% CO_2_ environment, all cells were harvested into flow cytometry tubes. After one wash with PBS, all samples were then incubated with anti-rat CD71-PE-conjugated antibody (a marker for activated and dividing T cells) for 20 minutes in dark on ice. Samples were then washed with PBS and T cell proliferation was measured by using flow cytometry analysis for CD71 expression. The average of the triplicate samples was recorded for each heart (n = 3 hearts/group/condition).

### Statistics

Data were analyzed using Student’s t-test (for comparison of cryoinjury and sham groups) or the analysis of variance followed by Newman-Keuls post hoc test to establish significance (p<0.05) among groups for all other data. Data are reported as means ± SEM.

## Results

**Fig 1:**
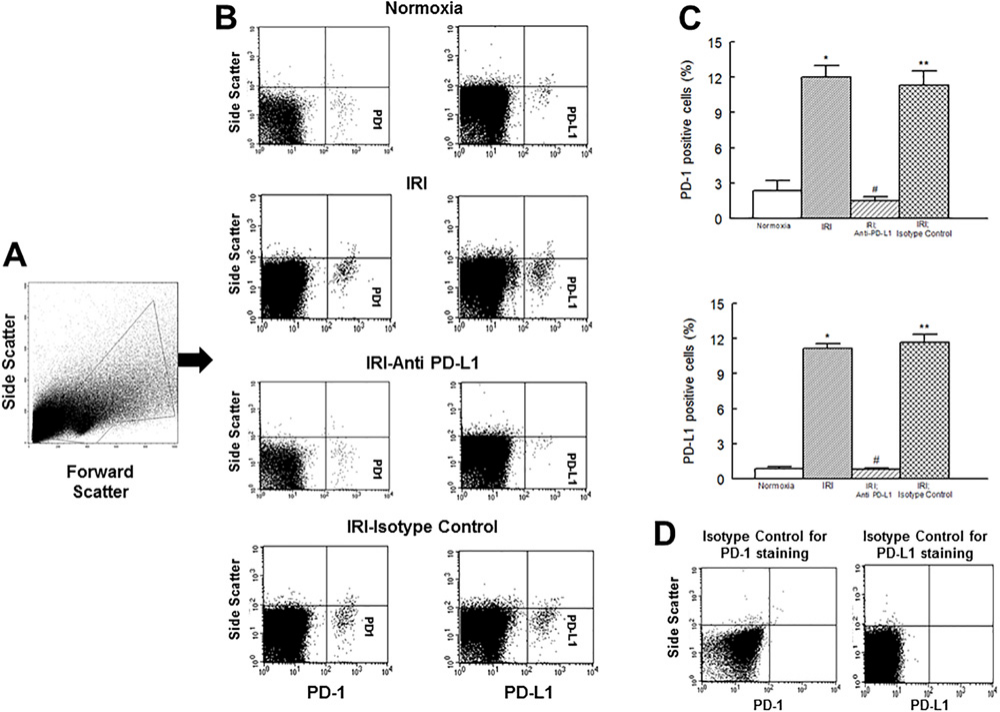
Flow cytometry profile of cardiac cell preparations outlining gated events is shown under panel A. Representative flow cytometry dot plots, under panel B, show increased expression of PD-1 and PD-L1 in cardiac cell preparations of ischemic-reperfused hearts compared to their normoxic controls, effects which were reversed by anti-PD-L1 antibody, but not by isotype matching antibody, treatment. Under panel C, bar graphs show percent of cells which were positive for either PD-1 or PD-L1; data are means ± SEM; n = 6 hearts/group for normoxic control, IRI and IRI; Anti-PD-L1 and n = 4 hearts for IRI; isotype matching control. Also shown under panel D are representative plots of staining isotype controls from FACS analysis for PD-1 and PD-L1 for gating purpose. IRI: Ischemia Reperfusion Injury. * p<0.05 compared to the normoxic group. ** p<0.05 compared to normoxia or IRI; Anti-PD-L1 group. # p<0.05 compared to IRI or IRI; isotype control group.

**Fig 2:**
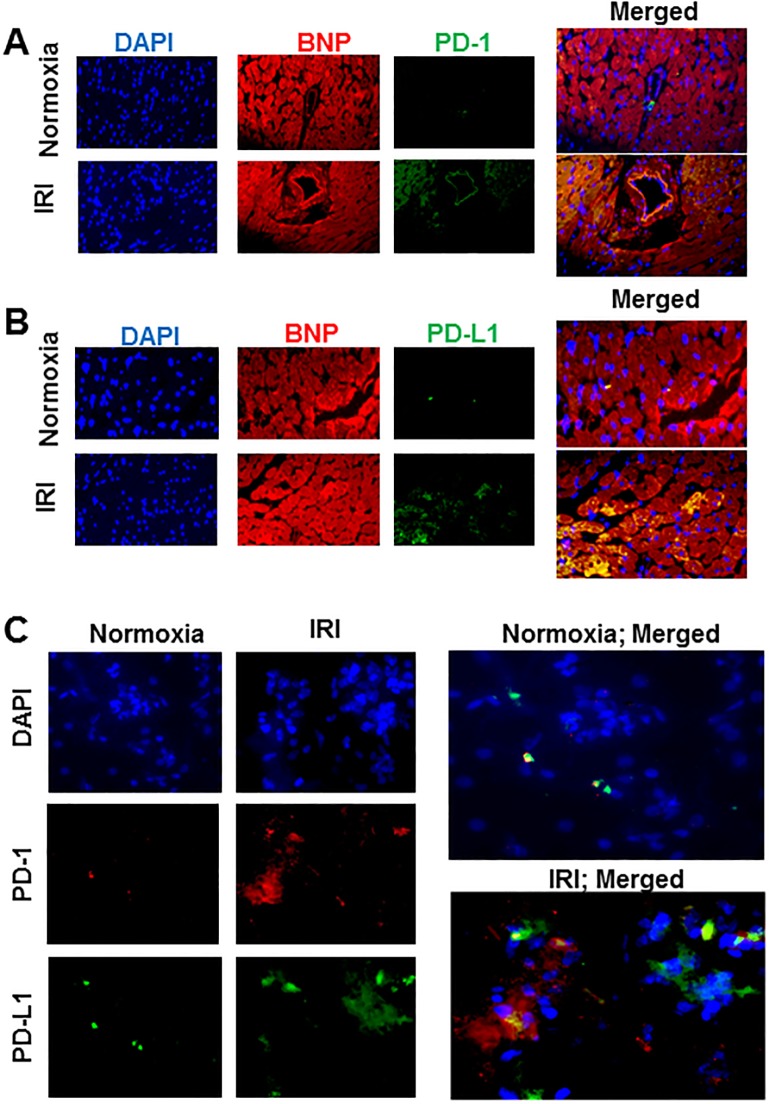
Panels show representative immunofluorescent images for DAPI (a nuclear marker), brain natriuretic peptide (BNP, a cardiomyocyte marker) and PD-1 (panel A) or PD-L1 (panel B) in normoxic and ischemic-reperfused hearts. Merged images in each panel show co-localization of PD-1 or PD-L1 with BNP indicating cardiomyocyte expression of PD-1 or PD-L1. Panel C shows representative images for PD-1 and PD-L1 immunofluorescent staining, as well as merged images, using cytospin preparations from normoxic and ischemic-reperfused hearts. Images are representative of 3 hearts for each condition. IRI: Ischemia Reperfusion Injury; DAPI: 4’, 6-diamino-2-phenylindole.

Fig 1 shows that normoxic hearts expressed low levels of both PD-1 and PD-L1. However, in response to an IR insult, cardiac expressions of both PD-1 and PD-L1 were markedly increased, an effect essentially abrogated in ischemic-reperfused hearts which were treated with the PD-L1 blocking antibody, but not isotype matching antibody, prior to the induction of the ischemic insult. In order to determine whether cardiomyocytes are a source of PD-1 and PD-L1, immunofluorescent studies were carried out using BNP as a marker of cardiomyocytes. Fig 2 shows that cardiomyocytes indeed express both PD-1 and PD-L1 as revealed by merged images of BNP (red fluorescence) with either PD-1 or PD-L1 (green fluorescence) producing yellow fluorescence (Fig 2A and 2B). We next explored whether PD-1 and PD-L1 are primarily expressed on the same cardiomyocytes by carrying out immunofluorescent studies using cytospin preparations. As shown by merged images under panel C of Fig 2, PD-1 and PD-L1 appear to be expressed by different populations of cardiac cells.

**Fig 3:**
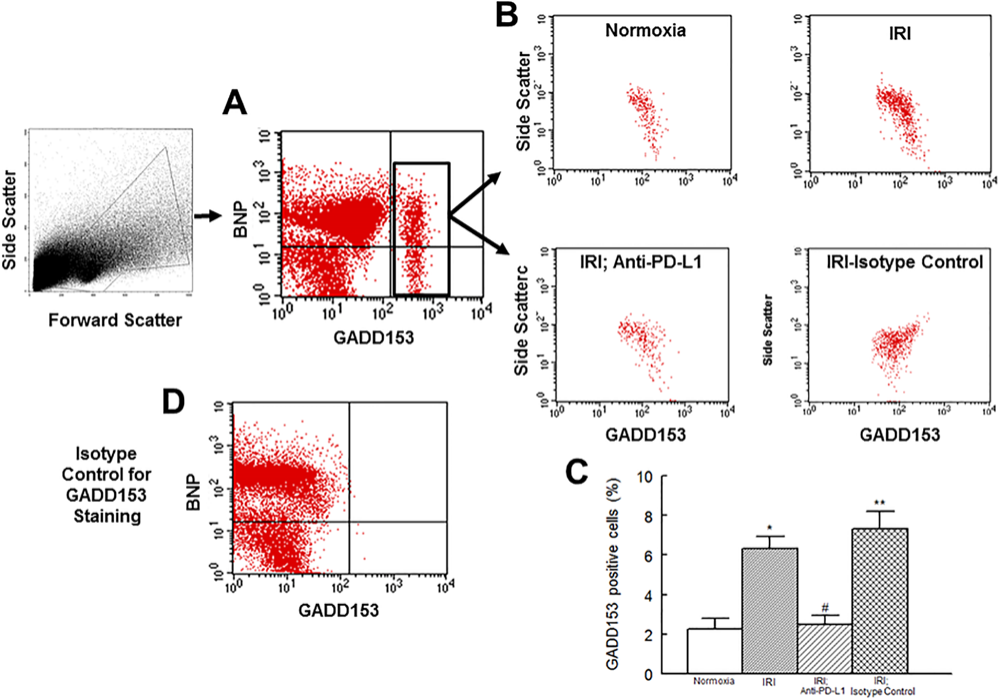
Under panel A, flow cytometry profile of cardiac cell preparation is shown along with a representative dot plot showing the expression of GADD153 in cardiac cell preparation; BNP was used to differentiate cardiomyocytes from other cardiac cells. Under panel B, representative dot plots are shown for GADD153+ cardiac cells from experimental groups. On the other hand, bar graphs, under panel C, show percent of cardiac cells which were positive for GADD153; data are means ± SEM; n = 6 hearts/group for normoxic control, IRI and IRI; Anti-PD-L1 and n = 4 for IRI; isotype matching control. Also shown, under panel D, is the staining isotype control for GADD153 from FACS analysis for gating purpose. IRI: Ischemia Reperfusion Injury. * p<0.05 compared to the normoxic group. ** p<0.05 compared to normoxia or IRI; Anti-PD-L1 group. # p<0.05 compared to IRI or IRI; isotype control group.

**Fig 4:**
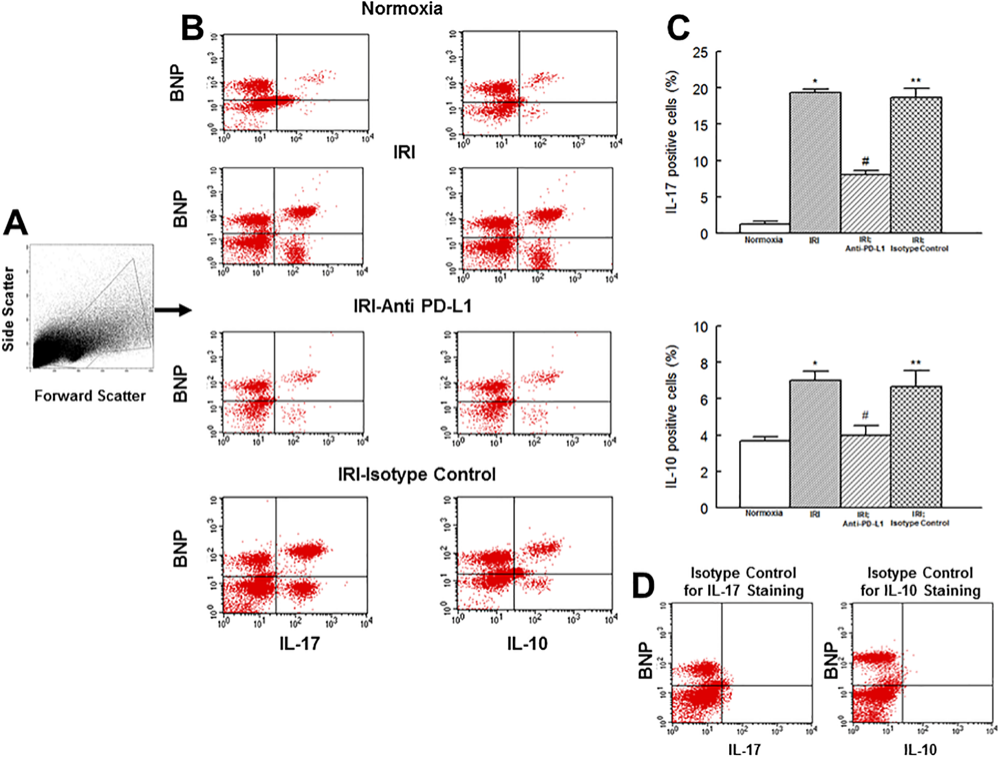
Flow cytometry profile of cardiac cell preparations outlining gated events is shown under panel A. Under panel B, representative scatter plots for interleukin (IL)-17 and IL-10 positive cells in heart cell preparations are shown for experimental groups. Bar graphs show percent of cells which were positive for either IL-17 or IL-10 (panel C); data are means ± SEM; n = 6 hearts/group for normoxic control, IRI and IRI; Anti-PD-L1 and n = 4 for IRI; isotype matching control. Also shown under panel D are representative plots of staining isotype controls for IL-17 and IL-10 from FACS analysis for gating purpose. IRI: Ischemia Reperfusion Injury. * p<0.05 compared to the normoxic group. ** p<0.05 compared to normoxia or IRI; Anti-PD-L1 group. # p<0.05 compared to IRI or IRI; isotype control group.

GADD153 is believed to regulate inflammation and cell death [16–18, 20]. Thus, we assessed its expression levels and those of IL-17 and IL-10 in the context of mitochondrial status and cell death. Fig 3 shows that GADD153 positive cells are significantly increased in hearts subjected to an IR insult and that treatment with PD-L1 blocking antibody, but not isotype matching antibody, reduces GADD153 expression to the level of the normoxic heart. Assessment of cytokine positive cells showed a very pronounced increase (about 16 fold) in the percent of IL-17 positive cells but a mild increase (about 55%) in IL-10 positive cells in hearts subjected to the IR insult compared to their normoxic controls (Fig 4). Importantly, treatment of ischemic-reperfused heart with the PD-L1 blocking antibody, but not isotype matching antibody, reduced levels of both IL-17 and IL-10 positive cells towards those of normoxic hearts but a significant difference persisted for IL-17 (Fig 4).

**Fig 5:**
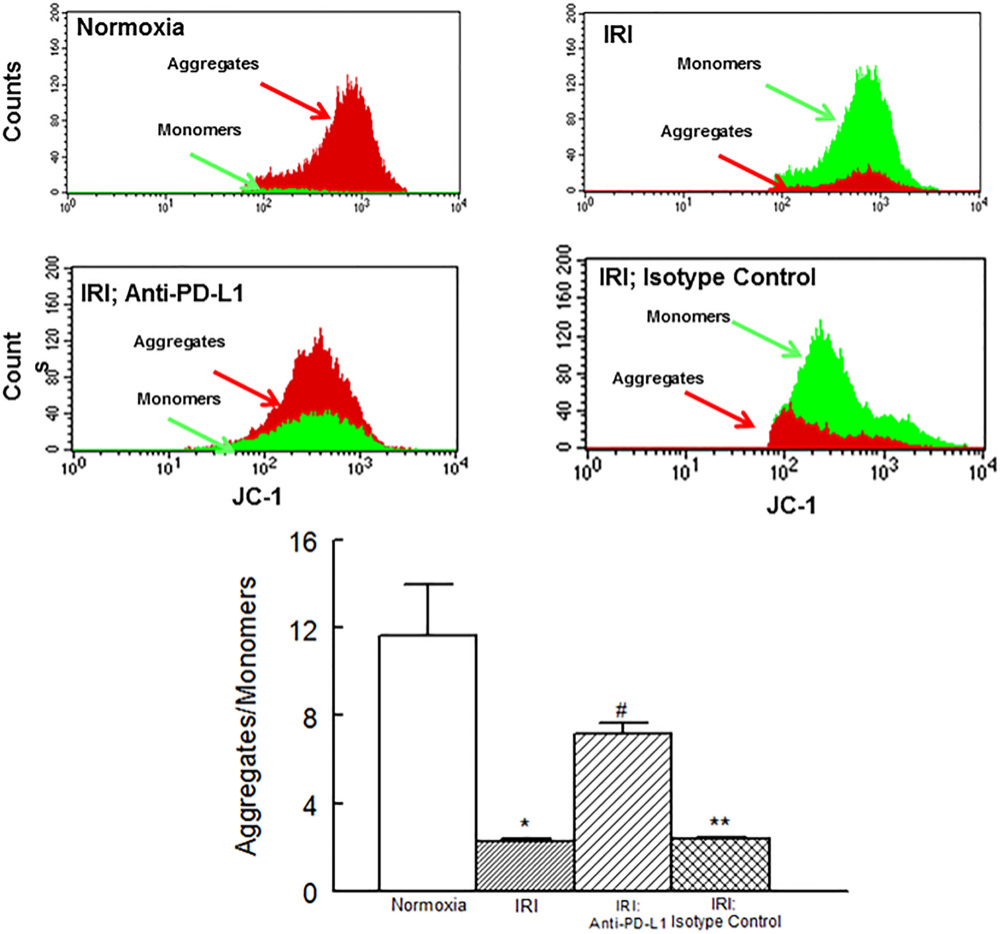
Panels show representative histograms for JC-1 monomers and JC-1 aggregates in cardiac cell preparations while bar graphs show the ratio of JC-1 aggregates to monomers for experimental groups. Data are means ± SEM; n = 6 hearts/group for normoxic control, IRI and IRI; Anti-PD-L1 and n = 4 for IRI; isotype matching control. IRI: Ischemia Reperfusion Injury. * p<0.05 compared to the normoxic group. ** p<0.05 compared to normoxia or IRI; Anti-PD-L1 group. # p<0.05 compared to IRI or IRI; isotype control group.

**Fig 6:**
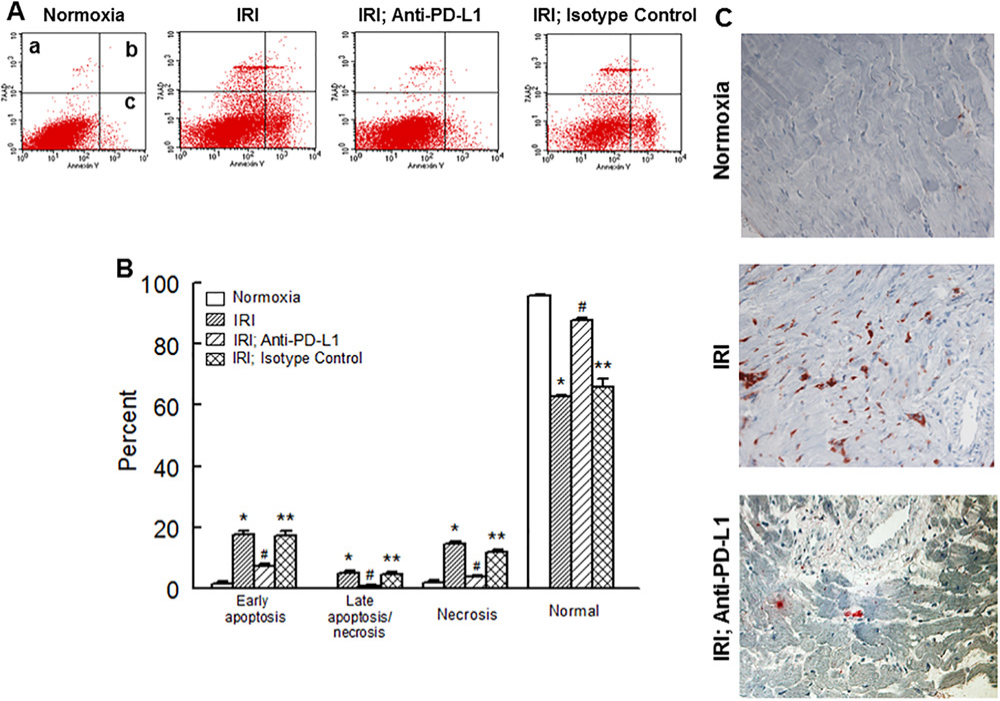
Representative dot matrices for apoptotic and necrotic cell death of experimental groups are shown under panel A while bar graphs (panel B) show percent of each type of damaged/dead or normal cells for each group. Data are means ± SEM; n = 6 hearts/group for normoxic control, IRI and IRI; Anti-PD-L1 and n = 4 hearts for IRI; isotype matching control. Also shown are representative caspase 3 immunostaining for experimental groups (panel C). 400x. 7AAD: 7-Amino Actinomycin D. a: necrotic; b: apoptotic/necrotic; c: early apoptotic. IRI: Ischemia Reperfusion Injury. * p<0.05 compared to the normoxic group. ** p<0.05 compared to normoxia or IRI; Anti-PD-L1 group. # p<0.05 compared to IRI or IRI; Isotype control group.

Fig 5 shows that an IR insult was associated with marked decrease in JC-1 aggregates compared to monomers suggestive of decreased mitochondrial membrane potential. Treatment with the PD-L1 blocking antibody, but not isotype matching antibody, significantly reduced the JC-1 aggregates/monomers ratio although a significant difference persisted compared to normoxic hearts. Fig 6, panel A, shows representative dot matrices for experimental groups showing necrotic (7-AAD+; a), necrotic/apoptotic (7-AAD+/Annexin V+; b) and early apoptotic (Annexin V+; c) cells while bar graphs, panel B, show mean values for each mode of cell damage for each group. Induction of an IR insult was associated with significant increase in each type of cell death thereby significantly reducing the percent of normal cells compared to the normoxic hearts. Treatment with the PD-L1 blocking antibody, but not isotype matching antibody, significantly reduced each form of cell death towards the normoxic control group although early apoptotic cell death remained significantly higher compared to the normoxic group. As a result, the percent of normal cells was significantly higher than the untreated ischemic-reperfused hearts but lower than normoxic hearts. Fig 6C shows representative caspase 3 immunostaining for experimental groups indicating marked increase in caspase 3 immunostaining for hearts subjected to an IR insult compared to the normoxic hearts and its reversal with PD-L1 blocking antibody.

**Fig 7:**
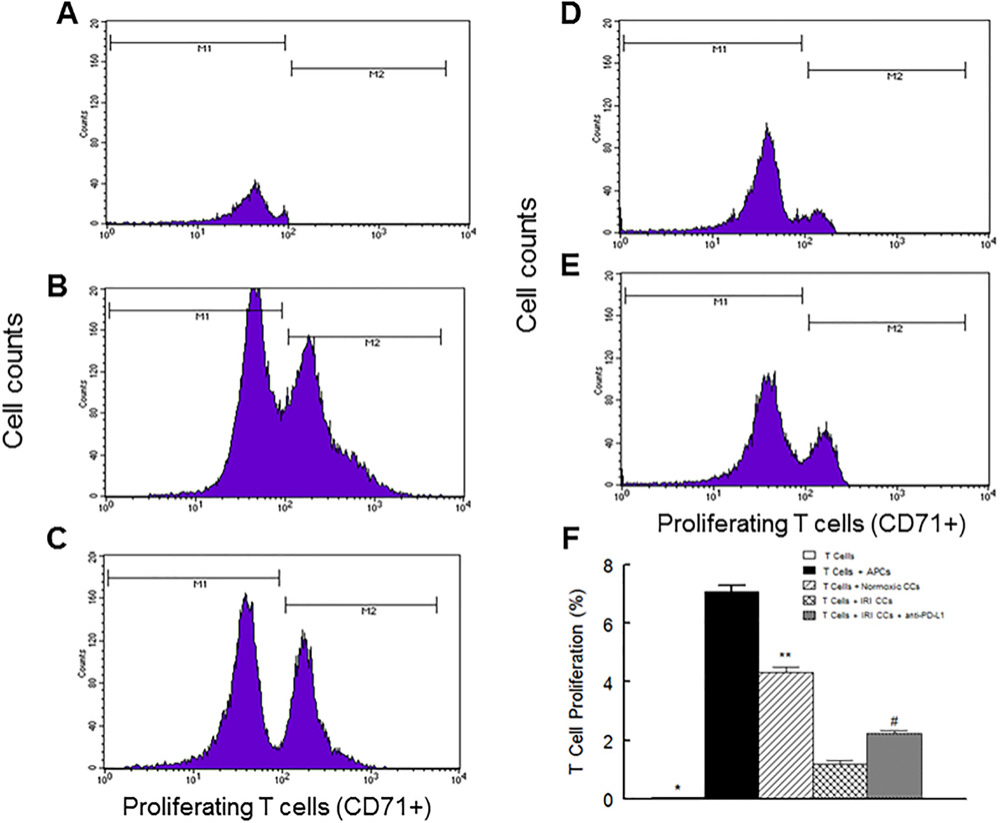
Panels A-E show representative histograms for proliferative pattern of T lymphocytes (i.e., CD71+). Panel A shows T lymphocytes alone while panel B shows T lymphocytes co-cultured with antigen presenting cells (APCs). On the other hand, panel C shows the pattern for co-culturing of T lymphocytes with normoxic cardiac cells (CCs) while panels D and E show patterns for T lymphocyte co-cultured with ischemic-reperfused CCs in the absence and presence of the PD-L1 blocking antibody, respectively. Panel F shows the percent of proliferating T lymphocytes under each condition; data are average of the triplicate samples for each heart (n = 3 hearts/group/condition). IRI: Ischemia Reperfusion Injury. * p<0.05 compared to other groups. ** p<0.05 compared to APCs or ischemic-reperfused CCs conditions. # p<0.05 compared to their untreated counterparts.

Given marked upregulation of PD-1/PD-L1 pathway in ischemic-reperfused cardiac cells, we next explored the influence of these cells on the proliferative capacity of T lymphocytes. Fig (7A–7E) shows representative histograms while panel F shows the percent of proliferating T lymphocytes (i.e., CD71+) under each condition. As expected, co-culturing of antigen presenting cells (APCs) with T lymphocytes resulted in remarkable proliferation of T cells (panel B vs. A; panel F). Interestingly, co-culturing of normoxic cardiac cells with T cells also caused a marked increase in proliferation of T cells (panel C; panel F) but the effect was significantly less than that observed for co-culturing of T cells and APCs (panel C vs. B; panel F). On the other hand, co-culturing of ischemic-reperfused cardiac cells with T cells markedly reduced proliferation of T lymphocytes compared to normoxic cardiac cells (panel D vs. C; panel F), an effect which was partially reversed by inclusion of PD-L1 blocking antibody in the assay medium (panel E vs. D; panel F).

**Fig 8:**
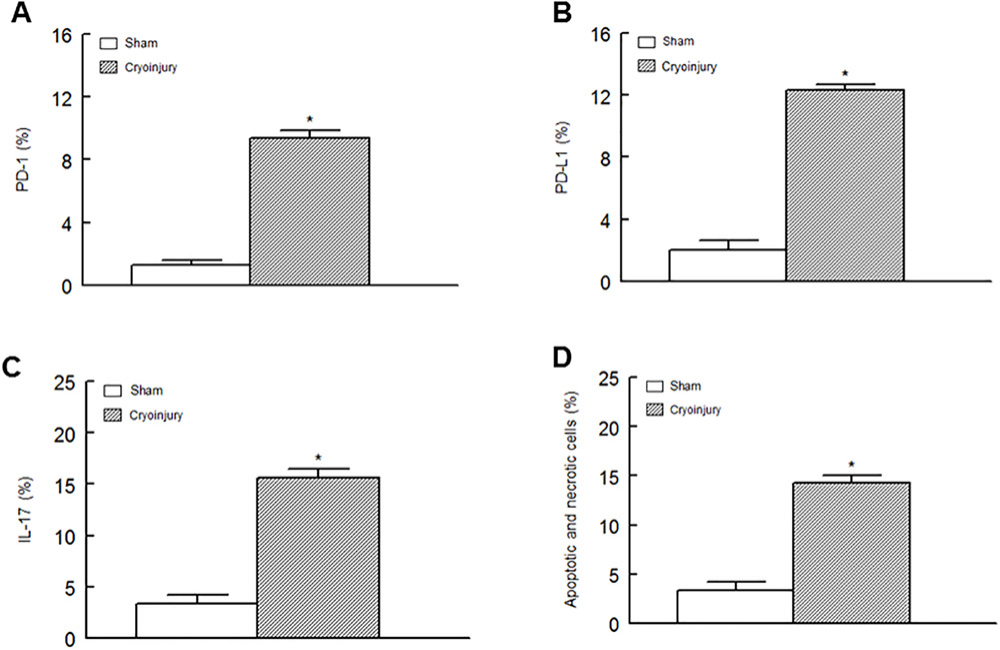
Hearts of male mice were subjected to cryoinjury or sham operation as described under Methods. Thereafter, cardiac cells were subjected to flow cytometry studies which revealed significant increases in PD-1, PD-L1 and IL-17 positive cells (panels A-C, respectively) in association with significant increase in apoptotic and necrotic cell death (panel D). Data are means ± SEM of n = 4 sham and n = 7 cryoinjury animals. * p<0.05 compared to the sham group.

As an initial step in establishing the *in vivo* relevance of PD-1/PD-L1 pathway in cardiac injury, subsequent studies utilized the cryoinjury model of myocardial infarction. Cryoinjury was associated with significant increase in GADD153 positive cells (9.4 ± 0.6 vs. 2.7 ± 0.3%; cryoinjury vs. sham, respectively). Further, as shown in Fig 8, hearts of mice which were subjected to cryoinjury displayed significant increases in percent of cardiac cells which were positive for PD-1, PD-L1 and IL-17 (panels A-C, respectively) in association with significant apoptotic/necrotic cell death (panel D) compared to hearts of sham-operated mice.

## Discussion

The present study indicates that an IR insult upregulates cardiomyocyte expression of PD-1 and PD-L1 in association with increased GADD153 expression and promotion of a proinflammatory environment culminating in disruption of ψ_m_ and marked necrotic/apoptotic cell death. Importantly, treatment with the PD-L1 antibody, but not isotype matching antibody, largely reversed the aforementioned changes in the ischemic-reperfused hearts. These observations indicate that upregulation of cardiac PD-1/PD-L1 pathway, in response to an IR insult, is a major determinant of tissue injury in the isolated buffer-perfused heart. Importantly, ischemic-reperfused cardiac cells inhibit proliferative capacity of T lymphocytes, an effect partially reversed by PD-L1 blocking antibody, thereby substantiating the inhibitory immune regulatory role of PD-1/PD-L1 pathway. Thus, while corroborating the antigen presenting role of cardiomyocytes, these novel observations establish the remarkable ability of an IR insult to influence proliferative capacity of T lymphocytes. Importantly, upregulation of PD-1/PD-L1 pathway is also a feature of the cryoinjury model of myocardial infarction thereby establishing its in vivo relevance for the damaged heart.

The role of programmed death pathway in regulation of tolerance and autoimmunity is well-established [1–3]. This pathway is an important player in tumor biology as exemplified by the demonstration that interruption of the interaction of PD-L1 with PD-1 exerts antitumor activity [6,7, 25–29]. With respect to the cardiovascular system, disruption of PD-1/PD-L1 pathway causes a number of outcomes including myocardial inflammation in a model of T cell myocarditis, viral myocarditis, autoimmune dilated cardiomyopathy and accelerated graft arterial disease in cardiac allografts as well as exacerbation of cytotoxic T-cell-mediated myocarditis [2,3,11,14,30,31,32]. Interestingly, however, intragraft expressions of PD-1, PD-L1 and PD-L2 occur during development of cardiac allograft rejection and targeting of PD-1 promotes allograft survival [33]. Further, in a murine model of stroke, simulated by middle cerebral artery occlusion followed by reperfusion, PD-1 signaling is suggested to serve a protective role by limiting inflammation and neurologic deficits while PD-L1 signaling is shown to enhance central nervous system inflammation and infarct volume [8, 13]. Collectively, these observations clearly suggest context-specific roles for PD-1 and its ligand, PD-L1, in pathological settings.

Myocardial injury is associated with upregulation of endogenous inflammatory responses [16–18]. Relying on the suppressive role of programmed death pathway in immune and inflammatory processes, we conjectured that the pro-inflammatory condition prevailing in the ischemic-reperfused heart may result, in part, from downregulation of this pathway. Interestingly, however, expressions of PD-1 and PD-L1 were markedly increased in the ischemic-reperfused heart which was subsequently shown to be expressed by cardiomyocytes. Immunofluorescent studies indicate that PD-1 and PD-L1 are expressed on different populations of cardiac cells. Thus, while both paracrine and autocrine modes of actions are proposed for regulation of immune cells by the PD-1/PD-L1 pathway [34,35], our findings suggest primarily a paracrine mode of action for this pathway in the ischemic-reperfused heart.

Upregulation of cardiac PD-1/PD-L1 pathway was accompanied with a robust increase in expression of GADD153 and promotion of a pro-inflammatory environment as revealed by a marked increase in IL-17 positive cells but a mild increase in IL-10 positive cells; we recently showed that the cardiomyocyte is a major source of inflammatory cytokine generation in isolated ischemic-reperfused heart [16, 19]. Since GADD153 is a regulator of the inflammatory response, it is plausible that the association of cardiac PD-1/PD-L1 pathway with pro-inflammatory changes of the ischemic-reperfused hearts is linked to GADD153 expression. This notion is supported by the demonstration that treatment with the PD-L1 blocking antibody markedly reduces cardiac GADD153 expression in association with significant reduction in expression of cytokines. To our knowledge, this is the first demonstration that not only an IR insult upregulates endogenous cardiac PD-1/PD-L1 pathway but that there is seemingly a mechanistic link between activation of this pathway, GADD153 expression and promotion of a pro-inflammatory response. It is noteworthy that treatment with the PD-L1 blocking antibody also reduced cardiac expression of both PD-1 and PD-L1 to those of normoxic hearts. The reason for this observation remains to be established. Nonetheless, it is likely that the cardioprotective effect of anti-PD-L1 reduces the generation of danger signals which, in turn, may serve as the trigger for upregulation of PD-1/PD-L1 pathway. A prototypical danger signal is the high-mobility group box 1 protein and an IR insult to the heart is known to markedly increase its generation [5]. On the other hand, expression of PD-1 could be dependent on availability of functional PD-L1. Thus, blocking of the PD-L1 could, in turn, result in reduced expression of PD-1. Aside from these considerations, it is noteworthy that several studies have shown potential interaction between PD-L1 and CD28/CD80/CD86 [33,36]. Thus, it is likely that blockade of PD-L1 may elicit effects other than those attributable to interruption of PD-1/PD-L1 pathway, aspects which remain to be established.

Upregulation of PD-1/PD-L1 pathway was associated with disruption of the ψ_m_ as well as necrotic and apoptotic cell death in the ischemic-reperfused heart. One likely mechanism for this association could relate to the marked increase in GADD153 expression since knockdown of GADD153 confers cardioprotective effect [18]. On the other hand, the deleterious impact of PD-1/PD-L1 activation on T lymphocytes is attributed to inhibition of the prosurvival signaling pathway, namely, phosphotidylinositol 3-kinase (PI3K)/Akt pathway. The PI3K/Akt pathway is a prominent component of the reperfusion injury salvage kinase pathway. Accordingly, activation of PI3K/Akt leads to increased phosphorylation/inhibition of the glycogen synthase kinase 3β (GSK-3β); GSK-3β is a likely converging point for diverse signaling pathways that regulate the MPT pore and cell fate [3, 19]. Thus, it is likely that upregulation of PD-1/PD-L1 in the ischemic-reperfused heart leads to inhibition of PI3K/Akt/GSK-3β pathway, ultimately leading to induction of the MPT pore and consequent cell death. In this context, it is noteworthy that pharmacologic inhibition of GSK-3β also markedly reduces GADD153 expression in the ischemic-reperfused heart [3]. Thus, one can envision a scenario whereby activation of PD-1/PD-L1 pathway either directly or indirectly, through GSK-3β, leads to increased GADD153 expression and consequent tissue injury. The notion that GADD153 plays a major role in mediating the impact of PD-1/PD-L1 pathway on the ischemic-reperfused heart is supported by the observation that treatment with the PD-L1 blocking antibody preserves ψ_m_ and markedly reduces cell death. Nonetheless, it is noteworthy that while the anti-PD-L1 treatment abrogated GADD153 expression and cytokine expression, significant differences in JC-1 aggregates/monomers ratio and cell death (i.e., primarily in early apoptosis) persisted between the untreated and treated ischemic-reperfused heart. The reason for this observation likely relates to multiple and divergent mechanisms that regulate the MPT pore and cell death in the ischemic-reperfused heart [19].

Given the marked upregulation of PD-1/PD-L1 pathway in the isolated ischemic-reperfused cardiac cells, we conjectured that co-culturing of these cells with T lymphocytes would be of consequence for their survival/proliferative capacity. This notion is based on the well-established interaction of the PD-L1, expressed on APCs, with PD-1, expressed on T cells, culminating in their apoptosis of the latter [12]. Since the isolated heart is devoid of the immune cells, we used the MLR functional assay to explore the impact of ischemic-reperfused cardiac cells, displaying upregulation of PD-1 and PD-L1, on CD3+ lymphocytes. Interestingly, cells prepared from normoxic hearts increased proliferation of T lymphocytes suggestive of their ability to serve as antigen presenting cells. In this context, it is noteworthy that expression of class I antigen is observed for the heart and that endothelial cells are capable of serving an antigen-presenting role [11, 25]. As expected, ischemic-reperfused cardiac cells markedly reduced the percent of proliferating T lymphocytes compared to those of normoxic cells, an effect partially reversed by treatment with PD-L1 blocking antibody treatment. The reason for partial recovery of proliferative capacity of T cells may relate to expression of other potential players by the ischemic-reperfused cardiac cells, aspects which remain to be established.

The isolated heart and MLR studies are suggestive of an important role for cardiac PD-1/PD-L1 in the ischemic-reperfused rat heart. As an initial attempt to further establish the relevance of this pathway in cardiac tissue injury, we utilized the murine cryoinjury model of myocardial infarction. This model produces standardized infarcts and is suggested to more closely resemble infarcts in clinical conditions than the coronary ligation model [24]. Interestingly, similar to IR injury in the isolated rat heart, the in vivo cryoinjury model of the mouse heart also caused significant increases in percent of cardiac cells which were positive for PD-1, PD-L1, GADD153 and IL-17 in association with significant increase in apoptotic and necrotic cells. The observation of upregulation of PD-1/PD-L1 following two distinct injurious stimuli using two rodent strains strongly implicates a major pathogenic role for PD-1/PD-L1 pathway in myocardial tissue injury.

In conclusion, the present study shows an important role of PD-1/PD-L1 pathway in cardiac injury. The expression of PD-1 and PD-L1 by different populations of cardiac cells (e.g., cardiomyocytes) is suggestive of a paracrine mode of action in the ischemic-reperfused heart. Upregulation of PD-1/PD-L1 is associated with GADD153 expression, proinflammatory changes accompanied with disruption of ψ_m_ as well as apoptotic and necrotic cell death; these effects are blocked by disruption of the PD-1/PD-L1 pathway, resulting in improved cardiomyocyte survival. We also demonstrate that cardiomyocytes are capable of inducing T cell stimulation, a process that is diminished by IR injury, which may reflect the upregulated PD-1/PD-L1 signaling in these cells. Collectively, our observations suggest that upregulation of PD-1/PD-L1 in response to cardiac tissue injury may have countervailing effects on cardiomyocytes and the immune system, the balance of which determines the extent of cardiac injury. Thus, the challenge remains how to harness the beneficial effects of cardiac PD-1/PD-L1 activation while preventing/reducing its deleterious effects on cardiac cells (in myocardial infarction and conditions associated with ischemia-reperfusion injury to the heart such as coronary artery bypass and cardiac transplantation). Importantly, determination of the relevance of cardiac PD-1/PD-L1 pathway as a potential immunotherapeutic target to curtail T cell-mediated cardiac graft rejection and associated complications represent fertile grounds for future investigation.
